# Protocol Outlines for Parts 1 and 2 of the Prospective Endoscopy III Study for the Early Detection of Colorectal Cancer: Validation of a Concept Based on Blood Biomarkers

**DOI:** 10.2196/resprot.6346

**Published:** 2016-09-13

**Authors:** Louise Rasmussen, Michael Wilhelmsen, Ib Jarle Christensen, Jens Andersen, Lars Nannestad Jørgensen, Morten Rasmussen, Jakob W Hendel, Mogens R Madsen, Jesper Vilandt, Thore Hillig, Michael Klærke, Anna-Marie Bloch Münster, Lars M Andersen, Berit Andersen, Nete Hornung, Erland J Erlandsen, Ali Khalid, Hans Jørgen Nielsen

**Affiliations:** ^1^Department of Surgical GastroenterologyHvidovre HospitalHvidovreDenmark; ^2^Digestive Disease CenterBispebjerg HospitalCopenhagenDenmark; ^3^Institute for Clinical MedicineUniversity of CopenhagenCopenhagenDenmark; ^4^Gastro Unit, Section for GastroenterologyHerlev HospitalHerlevDenmark; ^5^Department of SurgeryHerning HospitalHerningDenmark; ^6^Department of SurgeryHillerød HospitalHillerødDenmark; ^7^Department of Clinical BiochemistryHillerød HospitalHillerødDenmark; ^8^Department of SurgeryHorsens HospitalHorsensDenmark; ^9^Department of Clinical BiochemistryHospitalsenheden VestHolstebroDenmark; ^10^Department of SurgeryRanders Regional HospitalRandersDenmark; ^11^Department of Public Health ProgrammesRanders Regional HospitalRandersDenmark; ^12^Department of Clinical BiochemistryRanders Regional HospitalRandersDenmark; ^13^Department of Clinical BiochemistrySilkeborg HospitalSilkeborgDenmark; ^14^Department of SurgeryViborg HospitalViborgDenmark

**Keywords:** colorectal neoplasms, genomics, epigenomics, transcriptome, proteomics, metabolomics, early detection of cancer

## Abstract

**Background:**

Programs for population screening of colorectal cancer (CRC) have been implemented in several countries with fecal immunochemical testing (FIT) as the preferred platform. However, the major obstacle for a feces-based testing method is the limited compliance that reduces the clinical sensitivity for detection of participants with non-symptomatic CRC. Therefore, research approaches have been initiated to develop screening concepts based on biomarkers in blood. Preliminary results show that protein, genetic, epigenetic, and metabolomic components may be valuable in blood-based screening concepts, particularly when combinations of the various components appear to lead to significant improvements.

**Objectives:**

The protocol described in this paper focuses on the validation of concepts based on biomarkers in blood in a major population screened by FIT.

**Methods:**

In Part 1, participants will be identified and included through the Danish CRC Screening Program comprising initial FIT and subsequent colonoscopy to those with a positive result. Blood samples will be collected from 8000 FIT-positive participants, who are offered subsequent colonoscopy. Findings and interventions at colonoscopy together with personal data including co-morbidity will be recorded. Blood samples and data will also be collected from 6000 arbitrarily chosen participants with negative FIT. In Part 2, blood samples and data will be collected from 30,000 FIT-negative participants three times within 4 years. The blood samples will be analyzed using various in-house and commercially available manual and automated analysis platforms.

**Results:**

We anticipate Part 1 to terminate late August 2016 and Part 2 to terminate late September 2022. The results from Parts 1 and 2 will be presented within 12 to 18 months from termination.

**Conclusions:**

The purpose of this study is to improve the efficacy of identifying participants with neoplastic bowel lesions, to identify false negative participants, to identify participants at risk of interval neoplastic lesions, to improve the compliance in screening sessions, and to establish guidelines for out-patient follow-up of at-risk participants based on combinations of blood-based biomarkers.

## Introduction

Colorectal cancer (CRC) represents the second leading cause of cancer-related death in Europe with a high social and economic impact on human health [1,2]. Population-based screening has been shown to reduce the incidence of CRC and lead to improved overall survival, presumably because the disease is diagnosed in precursor or early stages [3,4].

Direct colonoscopy is considered the gold standard for CRC screening: sensitivity and specificity are higher than 95% for diagnosing neoplastic lesions if performed by experienced colonoscopists [5]. However, the feasibility of direct colonoscopy as a screening method is challenged by capacity requirements, costs, and side-effects such as bleeding, arrhythmia episodes, and bowel perforation. Consequently, a focus has been on the development of methods to identify participants having increased risk of CRC in order to selectively offer colonoscopy. At present, fecal immunochemical testing (FIT) is probably the optimal test for identifying high-risk participants as it outperforms other fecal-based tests when it comes to usability, sensitivity, compliance, and costs [6]. FIT has some limitations, however; the positive predictive value of FIT for diagnosing CRC is in the range of 6% to 10% [7-9], and consequently greater than 10 colonoscopies have to be performed in order to diagnose one case of CRC. An additional drawback is the risk of interval cancer - CRC diagnosed in the timeframe between screening rounds mostly due to false negative FIT results. The predominant challenge is the association with low patient compliance in the range of 33% to 64% which reduces the clinical sensitivity significantly [9,10], resulting in a large proportion of undiagnosed CRC among participants who have not taken the test in addition to participants with a false negative result of FIT. An urgent need has therefore emerged to develop improved methods for early detection of CRC with high performance regarding sensitivity and improved compliance.

Major efforts have been invested in research making use of biological markers in blood to identify high-risk participants who should be offered subsequent colonoscopy. A blood sample is easy to retrieve and is far more acceptable for a screening population consisting mostly of healthy participants compared with fecal samples required for FIT [11,12]. Blood-based biological markers of interest are a heterogeneous group consisting of various proteins, nucleosomes, transcriptomes, metabolomes, circulating tumor-derived DNA, and microRNAs having shown association with early stages of CRC [13-17]. In particular, a combination of biomarkers, which results in a complementing performance, has shown promising results [13,14,18-21].

It has been shown that the presence of various benign diseases including diabetes, liver fibrosis, asthma, and chronic obstructive lung disease may cause elevated levels of specific biological protein and methylated gene markers associated with CRC [13]. Such associations with epigenetics, transcriptomics, and metabolomics have not yet been evaluated, but particular results will be presented shortly. Statistical and mathematical analyses have shown that such associations may be taken into account and thereby blood-based biomarkers may still identify participants at risk of CRC [17]. In addition, an individualized risk assessment evaluation (RAE) can be performed in order to identify participants with high risk of having CRC. This is done by combining subject-based data (ie, age, gender, body mass index, the presence of other specific diseases, etc) with the results of the biological marker analyses [17]. The mathematical model of the RAE is dynamic and can be improved by adapting data according to new findings of potential biological markers or other individualized data found to be relevant for the risk of having CRC.

At present, the established RAE model is undergoing validation in a major multicenter study (the Endoscopy II Project) [22]. In collaboration with academic and industrial partners various genetic and epigenetic molecules, proteins, and metabolites are presently being analyzed in collected blood samples. Based on results achieved from that study, a specific profile will be constructed consisting of the combination of analyzed biomarkers selected according to the statistical association with early stage CRC and high-risk adenomas. This panel will then be used to validate the designed RAE model.

The population of the Endoscopy II Project consists of participants undergoing diagnostic colonoscopy due to symptoms attributable to CRC; hence the validated RAE model found in the Endoscopy II Project is only applicable to such populations. However, if the RAE is to be implemented in future programs the screening population will comprise asymptomatic participants. Hence, it is an absolute requirement that the RAE model is validated in an asymptomatic population. The aim of the present Endoscopy III Project is to validate the RAE model in a population consisting of participants of the Danish screening program.

## Methods

### Part 1

Eligible participants are identified through the Danish CRC Screening Program that is based on an outreach FIT and subsequent offer of colonoscopy to participants with positive test results. The screening program was initiated in March 2014 and is offered to Danish citizens of 50 to 74 years of age. Screening is to be repeated every second year after the initial implementation period of 4 years. The study population consists of two sub-groups, in accordance with the FIT result; FIT-positive participants (hemoglobin [hgb] ≥ 100 ng/mL; OC-Sensor platform) and FIT-negative participants (hgb < 100 ng/mL: OC-Sensor platform).

Participants undergoing subsequent screening colonoscopies at Amager/Hvidovre, Bispebjerg, Herlev, Herning, Hillerød, Holstebro, Horsens, Randers, Silkeborg, and Viborg hospitals will be invited to participate in the project. The result of the positive FIT and booking information for colonoscopy will be forwarded to the screening participants. Simultaneously, an electronic letter with study information and an invitation to attend the Endoscopy III Project will be forwarded to the participants. The inclusion was initiated May 2014 and is planned to terminate by late August 2016. The scheduled number of participating FIT positive participants is set to 8000.

At the day of the colonoscopy an informed consent must be signed by the participants before inclusion into the project; specifically, the study participants also sign that they allow access to health and pathology files according to the Danish Health Act §43,1-3. An interview will be conducted to obtain personal and possible concurrent disease data, followed by blood collection prior to colonoscopy.

Blood samples of 90 mL will be collected from an antecubital vein with light stasis and subsequently handled and stored according to a validated standard operating procedure including pre-analytical and analytical aspects such as blood sampling, handling, storage, and the effects of freeze/thaw on biomarkers of interest (Multimedia Appendix 1). Specifically, the blood samples for plasma are centrifuged twice to reduce the contaminating effects of leucocyte- and platelet-derived granule proteins. All collection and storage tubes are marked with unique barcodes, which by 8 digits identify the hospital where the sample is collected (2 digits), identification of the subject (4 digits), and identification of the specific tube content (2 digits; serum, plasma or buffy-coat). The Freezer Works PC-based storage handling system is used to track every sample in the -80^o^C freezers, which are electronically surveyed and monitored 24 hours a day, 7 days a week.

Data including findings and interventions at colonoscopy will be registered in a locked Web-based database and continuous audit of the database will be performed ensuring correct data recordings. At the end of the study on-site audit sessions will be performed as well.

The FIT-negative participants are arbitrarily selected from the database of the Danish CRC Screening Program and will be invited to blood collection at the nearest participating hospital. The inclusion started August 2014 and is planned to terminate by late August 2016. A total of 6000 participants will be recruited. The informed consent, data collection, and blood collection, handling, and storage will be carried out according to the procedure for the FIT-positive participants.

The emerging results from the Endoscopy II Project will be used to identify the specific biomarker profile subsequently used in analyzing data for Part 1 of the Endoscopy III Project. The analysis will be carried out as shown in Table 1.

### Part 2

Limitations of FIT screening include suboptimal sensitivity that leads to false negative results, and thereby some participants with CRC or high-risk or medium-risk adenomas (Textbox 1) are not identified.

Due to the prolonged implementation of FIT screening in Denmark, the period between the first and second screening rounds is estimated to be 4 years; limited colonoscopy capacity will, however, extend the implementation period to about 5 years. Therefore, even low-risk adenoma participants, who far from always are identified by the FIT test, may develop either high-risk/medium-risk adenoma or even early CRC in that prolonged period. Therefore, Part 2 of the Endoscopy III protocol, initiated in March 2016, will include blood samples and data from 30,000 participants screened negative by the FIT testing. The 10 participating hospitals have the capacity to include these participants within 24 to 30 months. Similar to Part 1 FIT-negatives (hgb<100 ng/mL; OC-Sensor platform) the participants are identified and arbitrarily selected from the database of the Danish CRC Screening Program and will be invited to blood collection at the nearest participating hospital. Informed consent, data collection, and blood collection, handling, and storage will be carried out according to the procedure for Part 1 of the project.

At inclusion the participants accept to have blood and data collections after 2 and 4 years, respectively. The third blood and data collection will be approximated to the period just after the second FIT screening testing. All participants will be advised by electronic mail (e-boks in Denmark) 1 month before data and blood collections after 2 and 4 years, respectively.

**Table 1:** Determination of the following biomarkers is in progress or planned in samples from the Endoscopy II Project with national and international academic and biotech laboratories.

Biomarker	Technique or platform
Profile of 8-15 plasma proteins	The Architect Automated platform, Abbott, Chicago, IL, USA
Soluble urokinase plasminogen activator receptor-I (suPAR-I), suPAR-II, suPAR-III, total suPAR	In-house ELISA platforms, Finsen Laboratory, Copenhagen, Denmark
Galectin ligands	In-house ELISA platforms, MD Anderson Cancer Center, Houston, TX, USA
Cathepsins	In-house ELISA platforms, University of Slovenia, Ljubljana, Slovenia
Cell-free DNA	inhouse PCR Platform, Roskilde Hospital, Roskilde, Denmark
Cell-free, tumor-derived DNA	Platform under development, Skejby Hospital, Skejby, Denmark
Nucleosomes	Tecan-based in-house ELISA platforms, Volition, Namur, Belgium
Proteomes	Mass-spectrometry-based platform, primarily tandem LCMS, Applied Proteomics Inc., San Diego, CA, USA
Metabolomes	Raman Hyperspectral Imaging, ChemImage INC., Pittsburg, PA,USA
YKL-40	Manual commercially available ELISA platform, Herlev Hospital, Herlev, Denmark
Profile of 4 soluble coagulation proteins	Luminex platform, Fred Hutchinson Cancer Research Center, Seattle, WA, USA
Nuclear magnetic resonance (NMR)	NMR profiles, University of Copenhagen, Copenhagen, Denmark
Gas chromatography-mass spectrometry (GC-MS)	GC-MS profiles, University of Copenhagen, Copenhagen, Denmark

**Categories of the identified adenomas.**

Adenoma characteristics

- High-risk adenoma
  - Lesion ≥ 20 mm or
  - ≥ 4 lesions or
  - Resection by piece-meal
- Medium-risk adenoma
  - Lesion ≥ 10 mm, but ≤ 20 mm or
  - 3-4 lesions independent of size or
  - Tubulovillous or villous lesion or
  - High-grade neoplasia
- Low-risk adenoma
  - < 3 lesions (< 10 mm) or
  - Tubular lesion or
  - Low-grade neoplasia

### Parts 1 and 2

Intensified follow-up of all included participants will be performed electronically via existing health databases and/or participant-specific health files using the unique 10-digit computerized Central Personal Registration number given to all Danish citizens. Thereby no-one is lost to follow-up. Among the aims are evaluations of the risk of developing intra-bowel as well as extra-bowel neoplasia among those diagnosed with adenomas, other benign bowel lesions, and those without any lesions in relation to the biomarkers determined in the project. It has previously been indicated that blood-based biomarkers may be useful to identify such at risk participants [23]. Specifically, the 30,000 FIT-negative participants will be followed-up according to the same aims to identify those with presence of bowel neoplasia although the FIT result was <100 ng/mL and those at risk of developing interval bowel neoplasia during the 4 to 5 years between the two first screening rounds.

Based on a number of approved addendums to the protocol the exact FIT hemoglobin concentrations will, among other parameters, be included for everyone participating in the project. Thereby combinations of blood- and feces-based screening platforms may improve the entire screening concept [24]. Among the aims is the ability to evaluate associations between hemoglobin concentrations and CRC or high-risk adenoma, medium risk-adenoma, low-risk adenoma, non-neoplastic bowel lesions or clean colorectum. Thereby, the amount of screen colonoscopies may be reduced significantly and independently of the cut-point hgb level of 100 ng/mL restricted to those who are in urgent need.

### Statistical Analysis

Sample size calculations are based on results from previous published pre-screening studies [8]. It is expected that a total of 8000 participants with a positive FIT test will be included in the study. This sample size results in a coverage probability (exact) of more than 90% for a half width less than 5% in the determination of the sensitivity. It is estimated that 450 to 500 participants will be identified with CRC, 2200 to 2500 with adenomas separated into high-risk, medium-risk and low-risk (Textbox 1); the estimation is that 15% to 20% of these will be identified with high-risk adenomas.

In addition, 6000 FIT-negative patients will be included as controls; the aim is to evaluate a possible difference in biomarker levels and/or presence between FIT-positive and FIT-negative participants. The specific inclusion of 30,000 participants with FIT-negative results will be performed without pre-protocol calculation; presumably, the amount is sufficient to perform the calculations outlined above, in particular because blood and data collections are performed trice in 4 years.

The primary endpoint is the detection of CRC. Secondary endpoints are high-risk adenomas and other non-bowel cancers [23]. The model determined in the Endoscopy II Project will be assessed in this study, in particular with emphasis on the sensitivities, specificities, and the predictive value of the risk. In addition, statistical modeling will be considered using logistic regression analyses, with biomarkers log transformed (base 2); all analyses will be adjusted for age and gender, hospitals will be considered as a random effect. The biomarkers included in the final model will be restricted to combinations of specific biomarkers with the highest likelihood score. Results will be presented by the odds ratios with 95% confidence intervals and area under of the receiver operating characteristic curve. Model validation will be assessed using 10-fold cross-validation. The chosen model will be applied to the FIT-negative samples and compared to the FIT-positive samples using a linear model. A secondary analysis of the FIT-positive samples will be done using decision tree analysis (Adaptive Index Model) [25]. Database management will be done by a locked Web-based database and the statistical calculations will be performed using SAS (v9.4) and R (R Development Core Team, Vienna, Austria).

### Ethical Approval

All planned procedures are in accordance with the ethical standards of the institutional and national research committee and with the 1964 Helsinki declaration and its later amendments or comparable ethical standards.

The protocol for the Endoscopy III Project has been approved by the Regional Ethics Committee (H-4-2013-050) and the Danish Data Protection Agency (2007-58-0015/HVH-2013-022).

## Results

We anticipate Part 1 to terminate late August 2016 and Part 2 to terminate late September 2022. The results from Parts 1 and 2 will be presented within 12 to 18 months from termination.

## Discussion

The primary purpose of Part I of the prospective Endoscopy III Project is to validate the RAE model in an asymptomatic population undergoing FIT screening for CRC. While the study is not a direct comparison between FIT and RAE because the population is selected from the Danish CRC Screening Program, and the included participants have either a positive or a negative FIT result, the results may indicate performance status of the RAE model. Another possible outcome is an indication of a complementary performance of the RAE model and FIT. The outcome may lead to future changes in the structure of implemented screening concepts to include a blood test with a possible supplementary feces test or visa-versa, or to offer the participant a choice between the two test modalities.

Most of the included participants in the present study are healthy and asymptomatic but achieved results are not directly referable to a possible screening population because of selection based on positive or negative FIT; hence, a higher or lower prevalence, respectively, of premalignant and malignant large bowel lesions in the two sub-populations is expected compared to the actual prevalence in the Danish population. The benefit of including the two sub-populations in the study leads to the possibility of comparing biomarker levels and/or the presence in high and low prevalence populations.

In Part 2 of the project, blood samples and data from the participants with negative FIT result may lead to information of how to identify those who either have a present or are at risk of developing interval neoplastic lesions. These participants are currently offered a second screening within a scheduled 4-year period according to the Danish CRC Screening Program. Inclusion of blood collection three times within that period may lead to detailed information on the biomarker levels and/or presence compared with the determination at the first screening and the association to risk of having or developing a neoplastic lesion. In addition, the information on velocity of biomarkers in bowel neoplasia may be specifically valuable to establish a basis for future directions of out-patient visits offered to participants at certain risk of developing bowel neoplasia.

At present, FIT is probably the most commonly used screening test for CRC in Europe, and is also in the implementation phase in some American states and Canada, but the test is challenged by low compliance. The compliance to blood sampling in the included participants in the present study is expected to be high due to expectations of a general acceptability in the population to a blood sample compared to a stool sample [11,12]. A previous study has shown compliance to blood sampling among participants with symptoms of CRC referred to colonoscopy of 96% [13]. Participants enrolled in the present Endoscopy III Project have already committed to screening in performing FIT and have accepted a subsequent colonoscopy. Hence, compliance found in the present study may not be directly comparable to the Danish population in general, but can give an indication of compliance to a blood sample in a healthy, screen-relevant population.

The amount of 90 mL blood collected and processed is not necessary for the planned analyses (Table 1). The repository of blood samples may, however, form a solid basis for future projects on new and potentially improved biomarkers of the growth, invasion, and/or dissemination processes of CRC; some of these may either stand alone or be integrated in the RAE model resulting in an improved RAE. The Ethics Committee of the Capital Region of Denmark has approved an amendment to the primary protocol (amendment #43062) to take care of the bio-bank.

The personal information, including information about concurrent diseases collected at the time of inclusion, is registered in the locked database. This provides the opportunity to perform follow-up on the future course of participants diagnosed with CRC or other benign or malignant diseases. The Ethics Committee has approved the follow-up opportunity according to The Danish Health Act §43.1-3, which subsequently is granted by participant signatures at the Consent Declaration. This could lead to hypotheses regarding an association between the analyzed biomarkers in this study and the course/prognosis of CRC. It could also lead to an indication of association between the analyzed biomarkers and presence/course of other malignant diseases; maybe even malignant diseases outside the large bowel [23].

We are aware that the ideal and definite study for validating the RAE would be a study including the background population of Denmark with randomization to outreach FIT or blood collection with subsequent colonoscopy in order to compare the value of the two screening tests. The logistic requirements and cost of such a study would be a major task, and is at present not feasible based on local research opportunities. However, the Endoscopy III Project will contribute to evidence for the use of blood-based biomarkers in the RAE model for CRC screening. The performance of FIT and the blood-based RAE will be compared in both the FIT-positive and FIT-negative cohort with colonoscopy considered as the gold standard. A comparable and/or better performance of the blood-based RAE could ultimately contribute to the argument of planning and financing a comparative, nationwide clinical trial of RAE versus FIT. In recent years, molecular pathological epidemiology has evolved describing premalignant adenomas as a heterogeneous group of various subtypes composed of different combinations of genetic and epigenetic variations in close interaction with non-genetic exposures [26]. Through cellular and extracellular interactions, genetic and epigenetic variants of premalignant adenomas and/or CRC could possibly influence levels of blood-based biological markers in the RAE model. However, collection and pathological evaluation of tissue samples from participants undergoing polypectomy/biopsy of tumor in Part 1 of the study is at present not feasible.

## Abbreviations

CRC: colorectal cancer
FIT: fecal immunochemical test
hgb: hemoglobin
RAE: risk assessment evaluation
suPAR: soluble urokinase plasminogen activator receptor
