# Supplementary material for: Protocol Outlines for Parts 1 and 2 of the Prospective Endoscopy III Study for the Early Detection of Colorectal Cancer: Validation of a Concept Based on Blood Biomarkers
Source: JMIR Res Protoc. 2016 Sep 13;5(3):e182. doi: 10.2196/resprot.6346 (PMC5039335; doi:10.2196/resprot.6346)
Supplement: Supplementary file 1 [file resprot_v5i3e182_app1.pdf]

ENDOSCOPY III: BLOOD COLLECTION, PROCESSING AND STORAGE

All collections must be performed in endotoxin-, DNA'se- and RNA'se free tubes from Becton Dickinson (B&D). Processed serum, plasma and buffy-coat samples must be stored frozen at -80°C in storage tubes from Almeco A/S, Esbjerg.

Tubes and collection material are delivered from the study secretary at Hvidovre Hospital or directly from Almeco A/S, Esbjerg on request.

Needed material per blood collection, handling and storage:
1 tourniquet
1 butterfly collection needle
3 red top tubes for serum, 10 ml/tube – additive: coagulation enhancer
6 purple top tubes for EDTA plasma, 10 ml/tube
20 Almeco storage tubes, capacity 2 ml/tube
4 Almeco storage tubes, capacity 4.5 ml/tube
1 Almeco re-centrifugation tube, capacity 50 ml/tube
4-5 Almeco disposable pipettes, capacity 5 ml
1 Almeco electronic pipette
1 Almeco pipette cone, capacity 5 ml
1 Almeco pipette cone, capacity 40 ml
50 barcode labels (00 x 25, plus 01 – 25)

Label interpretation:
8 digits - Example: 46 0123 07 – 46 = site identification
- 0123 = number of participant
- 07 = indicates content in tube (serum)

Labels that end with 00 must be used to identify collection tubes, CRF of the subject, etc.

Blood collection:
Prepare a set of collection tubes, identification by 00 labels. In addition, please use 00 labels on the 50 ml tubes for re-centrifugation. Prepare a set of storage tubes – use the actual numbers to identify serum, plasma and buffy-coats:

XX YYYY 01 – 09: serum, 0.8 – 1.0 ml/tube
XX YYYY 10 - 12: buffy-coat – only the white-cell layer – no additives
XX YYYY 13 - 16: EDTA plasma, 4.5 ml/tube
XX YYYY 17 – 24: EDTA plasma, 0.8 – 1.0 ml/tube

Get the name and cpr.nr. of the study participant; identify the CRF by the 00 label.

Apply light tourniquet at one of the upper forearms to identify a vein, which can be used for puncture using the disposable "butterfly" needle. Initiate collection using the red top tubes, followed by the purple top tubes. The collection tubes must be turned upside/down 5-10 times manually - or placed on an electronic blood turning device.

The red top tubes for serum must be placed for approximately ½ hour to finalize the coagulation process. Thereby risk of hemolysis and fibrin clotting is reduced. In addition, hemolysis may be

reduced by using light stasis, puncture of the largest vein and by adjusting the intra-tube pressure in tubes that are not completed (decap/recap the red top rubber plug).

- All 9 collection tubes must be centrifuged at **3000 x G for 10 mins at 21°C.**
- ***Idea:*** *the 6 purple top tubes are centrifuged separately, while the 3 red top serum tubes are kept for coagulation. Subsequently, the 3 red top tubes may be centrifuged with the 50 ml Almeco re-centrifugation tube.*
- After initiation the centrifugation process must not be interrupted
- Preset brakes at 45 sec

NB: Hemolysis cannot always be prevented. In the event of hemolysis – these samples must be handled similarly to non-hemolyzed samples.

Subsequently:

PLASMA, purple EDTA-tubes:

- After centrifugation the plasma from the 6 tubes must be transferred to one Almeco 50 ml re-centrifugation tube
- At pipetting of plasma from the 6 purple tubes it's allowed to reach – but not touch the buffy-coat. In the event that white cells from the buffy-coat is touched and some transferred to the Almeco re-centrifugation tube – it's urgent that the white cell pellet in the bottom of the Almeco re-centrifugation tubes is not touched – keep the pipette 0.5 cm above the pellet.
- The Almeco re-centrifugation tube must be centrifuged at **3000 x G for 10 mins at 21°C.**

During the re-centrifugation process, please do the following:

Buffy-coat, purple EDTA-tubes:

- The buffy-coat (white cell layer between plasma and red cells) from 3 of the purple top tubes that have been centrifuged and the plasma transferred to the Almeco 50 ml re-centrifugation tube must be transferred to 3 Almeco 2 ml storage tubes – one buffy-coat in each tube. It does not matter whether the buffy-coat amount is one (1) ml. Therefore, do transfer limited numbers of red cells – it can't always be avoided.
- The 3 Almeco buffy-coat storage tubes must be marked with barcodes 10-12

NB: The buffy-coat layer may stick to the sides of the centrifuged collection tubes. Please use the 5 ml disposable pipette to detach the layer before pipetting the buffy-coat to the storage tube

Re-centrifuged EDTA plasma:

- Please transfer approximately one (1) ml to each of the 8 Almeco 2 ml storage tubes – barcodes 17 – 24.
- Subsequently, transfer 4.5 ml to one Almeco storage tube – capacity 4.5 ml – barcode 13
- Finally, transfer the remaining EDTA plasma to 3 Almeco storage tubes – capacity 4.5 ml – balance the amount equally in each storage tube – barcodes 14-16

SERUM, red top collection tubes (must be completely coagulated before centrifugation):

- Please transfer serum from the 3 red top collection tubes to 9 Almeco 2 ml storage tubes
- Balance the serum into the 9 storage tubes – at least 0.8 – 1.0 ml in each
- Due to blood for serum that's not completely coagulated, a fibrin clot may hinder transferral of serum to the storage tubes. In that event, please re-centrifuge the red top collection tubes at **3000 x G for 10 mins at 21°C.**
- Barcodes 1 – 9.

The storage tubes must be separated in to storage boxes as follows:

- Barcodes 01-09: Serum
- Barcodes 10-12: Buffy-coat
- Barcode 13 EDTA plasma – Fred Hutch
- Barcodes 14-16: EDTA plasma – MOMA
- Barcodes 17-24: EDTA plasma

The storage tubes must be kept at -80°C until shipment to Hvidovre for registration anf long-term storage

Additional instructions:

- The entire collection, handling and storage process must be finalized within 2 hours
- All samples that have been centrifuged must be separated within 30 mins
- Samples must not be kept in direct sunshine or in rooms with temps > 30°C
- The barcodes must be marked with green color.
- Mark the specific part of the database that includes the storage tubes – tick the numbers with serum, buffy-coat, EDTA plasma. Empty storage tubes must be discarded.
- Be sure that the barcodes are identical on all storage tubes and on the CRF (datasheet).
